# Genome-wide identification of essential genes in *Mycobacterium intracellulare* by transposon sequencing — Implication for metabolic remodeling

**DOI:** 10.1038/s41598-020-62287-2

**Published:** 2020-03-25

**Authors:** Yoshitaka Tateishi, Yusuke Minato, Anthony D. Baughn, Hiroaki Ohnishi, Akihito Nishiyama, Yuriko Ozeki, Sohkichi Matsumoto

**Affiliations:** 1 Department of Bacteriology, Graduate School of Medical and Dental Sciences, Niigata University, 1-757, Asahimachi-Dori, Chuo-ku, Niigata 951-8510 Japan; 2 Department of Microbiology and Immunology, University of Minnesota Medical School, 689 23rd Avenue S.E. Microbiology Research Facility, Minneapolis, 55455 MN USA; 3 Department of Laboratory Medicine, Kyorin University School of Medicine, Tokyo, Japan

**Keywords:** Bacterial genomics, Bacteriology

## Abstract

The global incidence of the human nontuberculous mycobacteria (NTM) disease is rapidly increasing. However, knowledge of gene essentiality under optimal growth conditions and conditions relevant to the natural ecology of NTM, such as hypoxia, is lacking. In this study, we utilized transposon sequencing to comprehensively identify genes essential for growth in *Mycobacterium intracellulare*. Of 5126 genes of *M. intracellulare* ATCC13950, 506 genes were identified as essential genes, of which 280 and 158 genes were shared with essential genes of *M. tuberculosis* and *M. marinum*, respectively. The shared genes included target genes of existing antituberculous drugs including SQ109, which targets the trehalose monomycolate transporter MmpL3. From 175 genes showing decreased fitness as conditionally essential under hypoxia, preferential carbohydrate metabolism including gluconeogenesis, glyoxylate cycle and succinate production was suggested under hypoxia. Virulence-associated genes including proteasome system and mycothiol redox system were also identified as conditionally essential under hypoxia, which was further supported by the higher effective suppression of bacterial growth under hypoxia compared to aerobic conditions in the presence of these inhibitors. This study has comprehensively identified functions essential for growth of *M. intracellulare* under conditions relevant to the host environment. These findings provide critical functional genomic information for drug discovery.

## Introduction

As highlighted by research on the origin of tuberculosis, mycobacterial infections have been one of the greatest threats to humans over the past 70,000 years^1^. Recently, increasing attention has been paid to nontuberculous mycobacteria (NTM) because NTM infections account for a considerable proportion of mycobacterial disease worldwide^2^. Contrary to *Mycobacterium tuberculosis*, NTM reside in natural environments such as water and soil as well as in human-residential environments such as the bathroom^3,4^. Infection with NTM is estimated to occur from such habitats to humans. In Japan, more than 80% of the etiological agents of NTM disease are *Mycobacterium avium-intracellulare* complex (MAC; the generic name of *M. avium* and *M. intracellulare*) and the incidence of MAC disease is rapidly increasing^5^. In addition, a recent report from India has shown that NTM were detected in nearly one-third of clinical samples from patients suspected to have pulmonary and extrapulmonary tuberculosis at a tertiary care center^6^. As such, the health impact of NTM is spreading worldwide but there has been little progress in strategies for prevention and therapy in decades.

Advancements in next-generation sequencing (NGS) technology and transposon mutagenesis system have enabled the development of transposon sequencing (TnSeq), a population tracking method for fitness profiling of functions on a genome wide scale in various genera of bacteria^7^. The resulting lists of essential genes can be explored for possible drug targets because inhibition of the corresponding pathways theoretically leads to the suppression of bacterial growth^8^. In *M. tuberculosis*, TnSeq has revealed genes required for fitness in optimal medium^9,10^, in minimal medium^11,12^, in response to CD4 T cell immunity^13^, and has elucidated genes associated with *in vivo* persistence^13^ and different susceptibilities to antibiotics^14^. In NTM, essential genes have been determined in *M. marinum*, the main host of which is poikilothermic animals such as fish, frogs and reptiles^15^. However, to our knowledge, such functional genomic studies have not yet been reported for the human pathogenic NTM.

We recently discovered that one of the etiological agents of MAC disease, *M. avium* subsp. *hominissuis* (MAH) forms a pellicle biofilm suspended at the air-liquid interface when cultured under hypoxia^16,17^, implicating a role of hypoxia in biofilm formation as an ecological adaptation, such as residing in natural water with limited aeration^18^ or in hypoxic granuloma *in vivo*^19^. Biofilm formation is considered to be an important phenomenon for bacteria to achieve long-term survival in harsh environments such as in nature and inside human hosts^20^. The microenvironments inside biofilms are known to be hypoxic because oxygen only penetrates approximately 50 μm into the biofilms^21^. In biofilms, bacteria produce various kinds of surface molecules and secreted proteins, and also form extracellular matrix to protect the bacterial community^22^. Such biofilms can be a source of infection, etiologically from the environments to human body, and microscopically from one focus to another in the infected organs like the lung and lymph nodes. Therefore, the elucidation of functions essential for biofilm formation is expected as an entry point for identifying markers for infection control.

In this study, we identified essential genes on a genome-wide scale by using TnSeq analysis. The identified list of genes included numerous virulence-associated genes, some of which were suggested to be promising drug targets that were more critical for bacterial survival under hypoxia than aerobic conditions. Moreover, the TnSeq data provide the information of the metabolic remodeling in hypoxic survival to form a pellicle. This study provides the basic database of gene essentiality in NTM, which enables us to deepen our understanding of NTM biology.

## Results

### Generation of Tn mutant library

**Figure 1:**
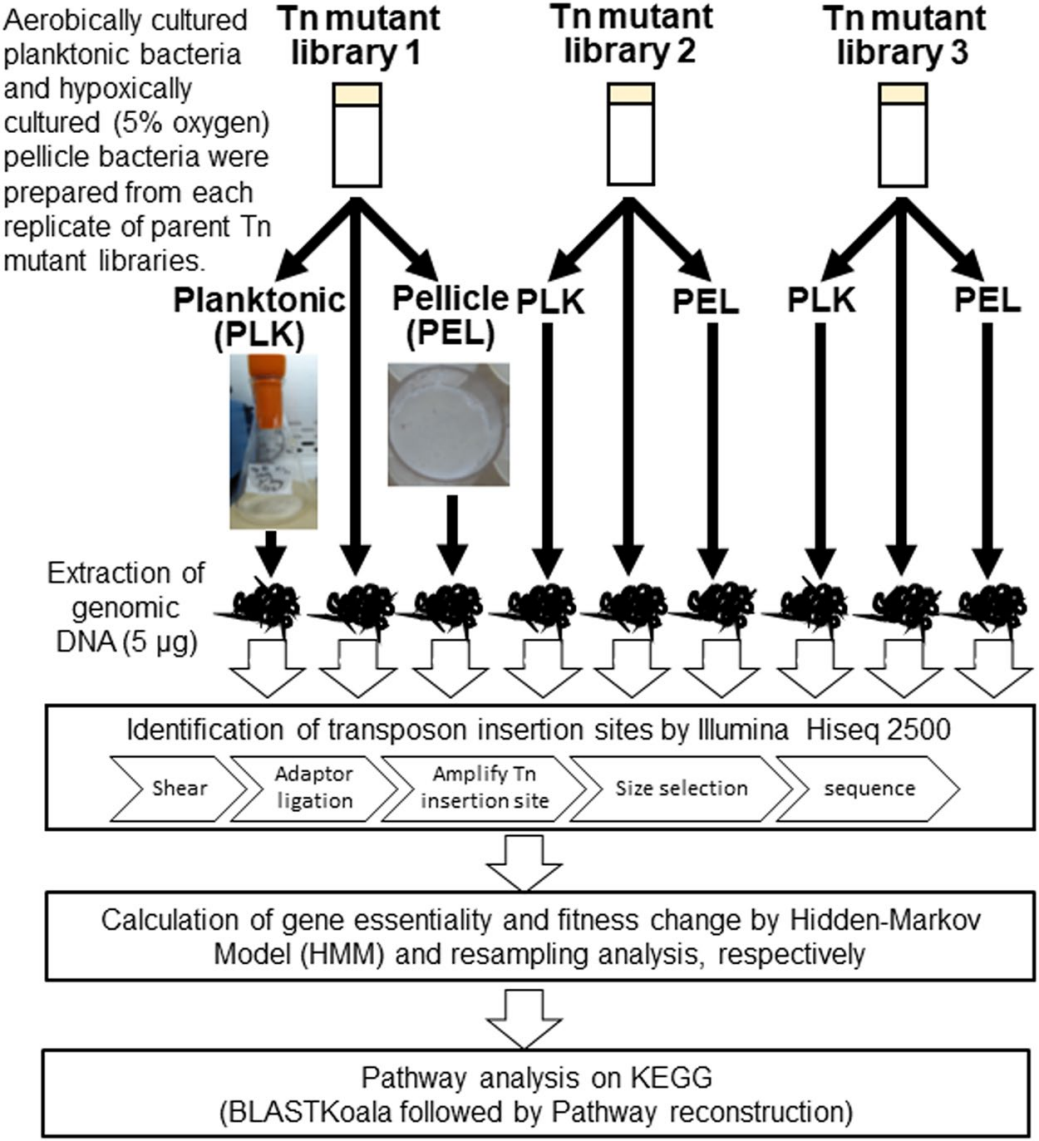
Flowchart of the experiment procedure.

**Figure 2:**
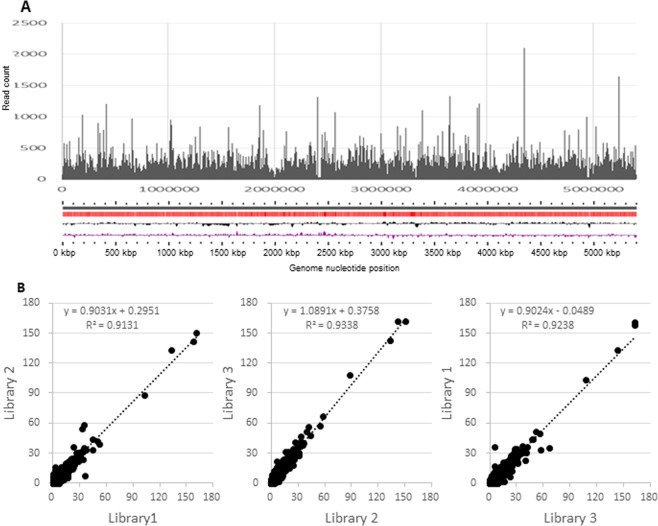
Overview of the transposon (Tn) insertion data produced by next-generation sequencing. (**A**) Distribution and density of transposon insertion on the ATCC13950 genome. Tn insertion reads (vertical black bars) are shown in the upper row. Coding sequence (CDS) (red), %GC plot (black, upward = above average, downward = below average) and GC skew (purple) are shown in the lower row. (**B**) Sequencing reproducibility. The graphs compare the number of insertion reads per gene between batches of the Tn mutant libraries used in this study.

The flowchart of this study is shown in Fig. 1. We constructed three saturated Tn mutant libraries by harvesting >1.7 × 10^5^ mariner transposon mutagenized colonies. As the *M. intracellulare* ATCC13950 genome (accession number: NC_016946.1) contains 64,293 TA sites, we anticipated that each library would show greater than 2.5-fold coverage per insertion. We performed TnSeq to obtain basic data on ATCC13950 essential genes. TnSeq yielded more than 2 million reads per sample. By Bowtie2 mapping, about 60% of the reads were aligned to the genome sequence (Table 1), which was a comparable mapping ratio to the previous report^9^. Out of the 64,293 TA sites present in the *M. intracellulare* ATCC13950 genome, the average number of TA sites targeted by the transposon was 32,697. We checked whether our Tn insertion system guarantees high reproducibility in each batch of experiment by comparing the number of the reads mapped to each gene with each Tn mutant library and found an excellent correlation (R^2^ > 0.9) between libraries (Fig. 2).

**Table 1:** Result of next-generation sequencing and mapping data.

Library	No. of sequence reads	No. (%) of aligned reads	No. (%) of insertion site flanking sequences hit in library	Average no. of reads/flanking sequence
Library 1	3,620,129	2,112,345 (58.4%)	31,792 (49.4%)	66
Library 2	2,299,076	1,329,326 (57.8%)	30,616 (47.6%)	43
Library 3	3,067,571	1,827,659 (59.6%)	35,682 (55.5%)	51

### Comparison of gene essentiality between *M. intracellulare* and other mycobacteria

**Figure 3:**
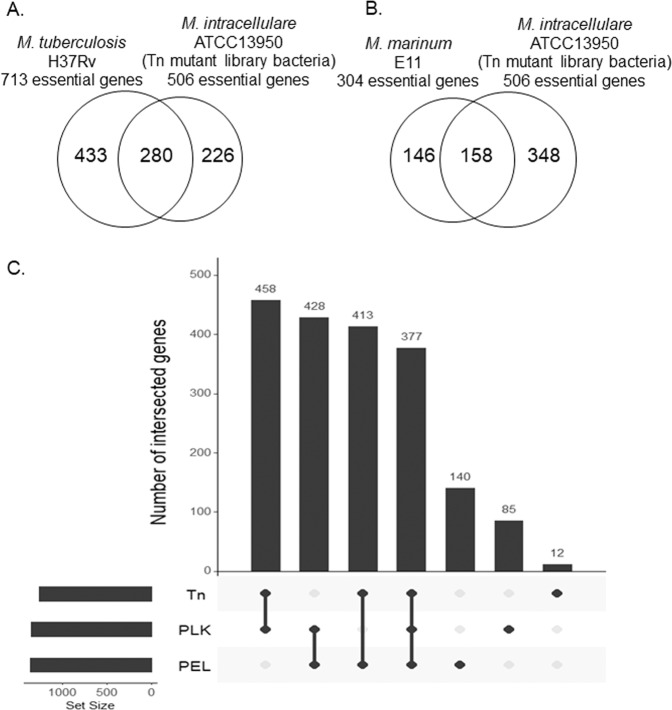
Relationship of the number of genes identified as essential by the Hidden Markov Model (HMM) analysis. (**A**) Venn-diagram between *M. tuberculosis* H37Rv and *M. intracellulare* ATCC13950. (**B**) Venn-diagram between *M. marinum* E11 and *M. intracellulare* ATCC13950. (**C**) UpSet plot between Tn mutant library bacteria, aerobically cultured planktonic bacteria (PLK) and hypoxically-cultured pellicle bacteria (PEL) in *M. intracellulare* ATCC13950.

After averaging the obtained read counts between the three replicates of Tn mutant libraries, we determined the essential genes by using the Hidden Markov Model (HMM), a transition probability method that can be applied on the read counts at the site and the distribution over the surrounding site, based on the assumption of potential data fluctuation on the series of data^23^. We found that 506 genes were determined as essential, where the mean likelihood of read counts was near-zero (Tables S1,S2). Of the 506 essential genes, 280 and 158 genes were shared with *M. tuberculosis* H37Rv (having a total of 2,187 homologous genes with *M. intracellulare* ATCC13950) and *M. marinum* E11 (having a total of 2,593 homologous genes with *M. intracellulare* ATCC13950), respectively (Figs. 3A,B, Table S3)^11,15^. The shared genes included genes of fundamental functions such as DNA replication (*dnaA, dnaN, gyrB, gyrA, dnaB, dnaG, polA, dnaE*, topoisomerase gene *topA*), RNA polymerase (*rpoA, rpoB, rpoC*), chaperone (*clpX, dnaK, groEL*), general secretion (Sec) system (*secA, secD, secE, secF, secY, yidC*), ribosomal proteins, tRNA biogenesis, lipid biosynthesis (fatty acid synthase *fas*, fatty acyl CoA synthase, β-ketoacyl ACP synthase *kas*, β-ketoacyl ACP reductase *inhA*), 2-oxocarboxylic acid metabolism (*idh1, leuB, leuC, leuD*), murein synthesis, amino acid synthesis (tryptophan, histidine) and heme synthesis (*hemB*). Furthermore, the gene essentiality of existing antituberculous drug targets was also demonstrated (Table 2), thus providing the microbiological validation of the combined use of antituberculous drugs for NTM disease treatment. Mycobacteria-specific genes such as mycolic acid-containing polyketide synthase gene *pks13* and trehalose monomycolate transporter gene *mmpL3*, which is the target gene of N′-(2-adamantyl)-N-[(2E)-3,7-dimethylocta-2,6-dienyl]ethane-1,2-diamine (SQ109), were also revealed to be essential. Meanwhile, ATCC13950-specific essential genes included genes of AAA family ATPase, several kinds of transporters such as metal transporters and ABC transporters, and several kinds of transferases and dehydratases. However, the majority of the remaining unshared genes were annotated as hypothetical (Table S3).

**Table 2:** Essential genes of *M. intracellulare* ATCC13950 corresponding to existing antituberculous drug targets.

Gene locus	Gene	Function	Target drug	Minimum inhibitory concentration
OCU_RS25020	*gyrB*	DNA topoisomerase (ATP-hydrolyzing) subunit B	fluoroquinolone	0.867 μM (levofloxacin)
OCU_RS25025	*gyrA*	intein-containing DNA gyrase subunit A	fluoroquinolone	
OCU_RS26150	*embB*	arabinosyltransferase	ethambutol	24.5 μM
OCU_RS26155	*embA*	arabinosyltransferase	ethambutol	
OCU_RS26175	*embC*	arabinosyltransferase	ethambutol	
OCU_RS40185	*inhA*	enoyl-[acyl-carrier-protein] reductase FabI	isoniazid/ethionamide	500 μM/1.92 mM (isoniazid/ethionamide)
OCU_RS41875	*dfrA*	dihydrofolate reductase	para aminosalicylic acid	4.08 μM
OCU_RS45570	*alr*	alanine racemase	cycloserine	98.0 μM
OCU_RS46230	*rpoB*	DNA-directed RNA polymerase subunit β	rifampin	11.9 nM
OCU_RS48685	*mmpL3*	membrane protein MmpL3	SQ109	37.8 μM

### Essential genes in aerobically-cultured planktonic bacteria and hypoxically-cultured pellicle bacteria in *M. intracellulare*

NTM is characterized by dual residence in natural environments and *in vivo* infection including humans. To reside in natural environments, tolerance to changes in ecological patterns has been emphasized, as is the case with biofilm formation^20^. The common environment under these conditions is hypoxia as suggested by low oxygen concentration in natural water, tuberculous granuloma and inside biofilms in biofilm-forming bacteria^18,19,21^. First, we confirmed that, similar to pellicle formation in MAH as we demonstrated previously^16^, *M. intracellulare* ATCC13950 formed a pellicle under an atmosphere of 5% oxygen (Fig. S1). After preparing aerobically-cultured planktonic (PLK) bacteria and hypoxically-cultured pellicle (PEL) bacteria from each replicate of the Tn mutant libraries (Fig. 1, Table S5, Fig. S2), we compared the profile of the essential genes of PLK and PEL bacteria with those identified in the Tn mutant libraries (Fig. 3C). Eighty-five genes were found to be essential specific to PLK bacteria and these included genes involved in glycolysis, such as pyruvate kinase, phosphoglycerate kinase and glyceraldehyde-3-phosphate dehydrogenase. This suggests the requirement of glycolysis to produce energy for the onset and maintenance of planktonic growth. By contrast, one hundred-forty genes were found to be essential specific to PEL bacteria and these included genes for phosphate transport and signaling complex proteins, phosphatidylinositol mannosyltransferase, nitrate and nitrite reductases, several polyketide synthases, glycine cleavage system,, nonribosomal peptide synthases, some ribosomal proteins, some mycothiol redox protein and type VII secretion system proteins of ESX-3 (Table S6). These findings are consistent with a response to phosphate limitation^24,25^, nitrogen deprivation^26^ and thioredoxin-related oxidative stress^27,28^. As discussed below, several of these genes also showed fitness costs during hypoxic exposure (Table S7).

### Gene requirements in pellicle bacteria in *M. intracellulare*

**Figure 4:**
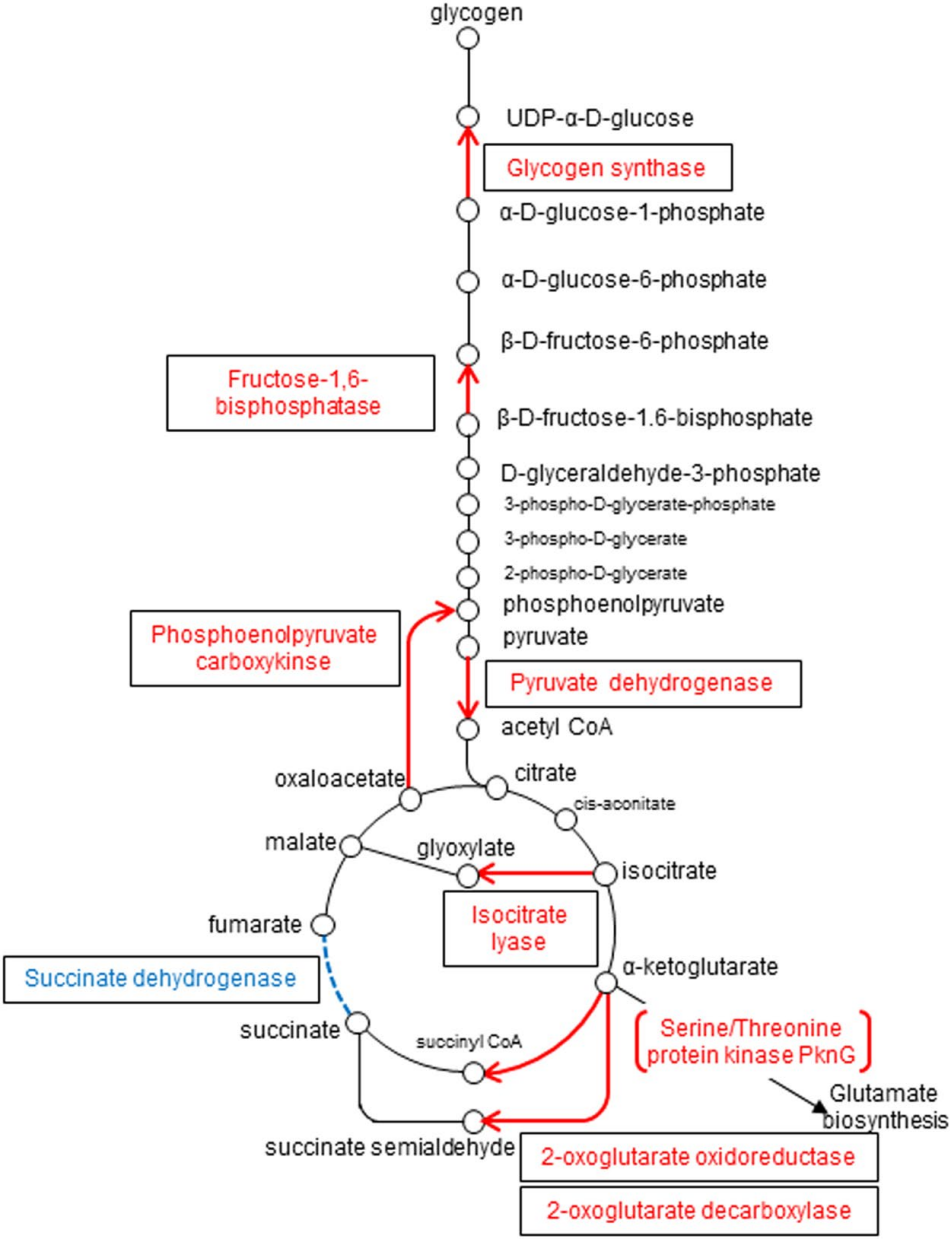
Preferential carbohydrate metabolism speculated by the TnSeq result. Gluconeogenesis, glyoxylate cycle and succinate production from α-ketoglutarate were estimated to be required under hypoxia. α-ketoglutarate is also a precursor of glutamate biosynthesis through the balancing of glutamine/glutamate by serine/threonine protein kinase PknG. By contrast, succinate dehydrogenase was estimated to play a minor role under hypoxia as shown by the fitness increase of succinate dehydrogenase flavoprotein subunit gene.

In addition to the gene essentiality in each ecological condition, fitness change is also an important factor for bacterial survival in various specialized environments. To evaluate the genes showing fitness change during hypoxic pellicle formation, we performed resampling analysis, a gene-based permutation model that calculates the difference between the sum of the read counts at each condition, performs 10,000 permutations and plots the observed differences as a histogram for determining the *P*-value^23^. Of 180 genes hit by resampling analysis, 175 showed significantly decreased fitness and the remaining 5 genes showed increased fitness, which resulted in the increase of the number of required genes during hypoxia compared to aerobic conditions (Table S8). The genes showing decreased fitness covered a wide range of metabolism such as carbohydrate, amino acid, fatty acid, cofactor, purine, cell wall synthesis, genetic information process, and various kinds of transporters. Of note, Tn insertions were significantly decreased in PEL bacteria in carbohydrate metabolism genes, especially gluconeogenesis (fructose-1,6-bisphosphate isomerase, pyruvate dehydrogenase), succinate production (α-ketoglutarate oxidoreductase, α- ketoglutarate decarboxylase) and glyoxylate cycle (isocitrate lyase) (Table S9). By contrast, Tn insertions in succinate flavoprotein subunit gene (OCU_RS48340) were significantly increased (Table S8). Mapping of these genes indicated the preferential gluconeogenesis and succinate production during hypoxic growth to form a pellicle, as suggested by previous research in *M. tuberculosis*^29–32^ (Fig. 4).

The genes showing decreased fitness also included virulence-associated genes in a wide range of metabolic pathways; i.e. synthesis of folate (*para-*aminobenzoate synthetase *pabB*, 4-amino-4-deoxychorismate lyase *pabC*)^33^ and F420 biosynthesis (coenzyme F420-0: L-glutamate ligase *fbiB*)^34^, cell wall metabolism of peptidoglycan (penicillin-binding protein A *pbpA*)^35^ and phosphatidylinositol (α-1,2-mannosyl transferase *pimE*)^36,37^, proteasome system (proteasome subunit α *prcA* and β *prcB*)^38^, two- component system (serine-threonine protein kinase *pknG*, potassium-transporting ATPase *kdpA*)^27,39,40^, type VII secretion system of ESX-3 (secretion-associated serine protease mycosin *mycP3*, PPE family protein, secretion protein *eccC3*)^41,42^ and ESX-5 (secretion-associated serine protease mycosin *mycP5*, integral membrane protein *eccD5*, secretion-associated protein *espG5*, secretion protein *eccC5*)^43^, mycothiol metabolism (D-inositol-3-phosphate glycosyltransferase)^27,28^, mycobacterial membrane protein L and respiratory-chain quinone metabolism (menaquinone-9 β-reductase) (Table S9)^44^.

### Validation of TnSeq fitness data by growth inhibition assay by using metabolic inhibitors

**Figure 5:**
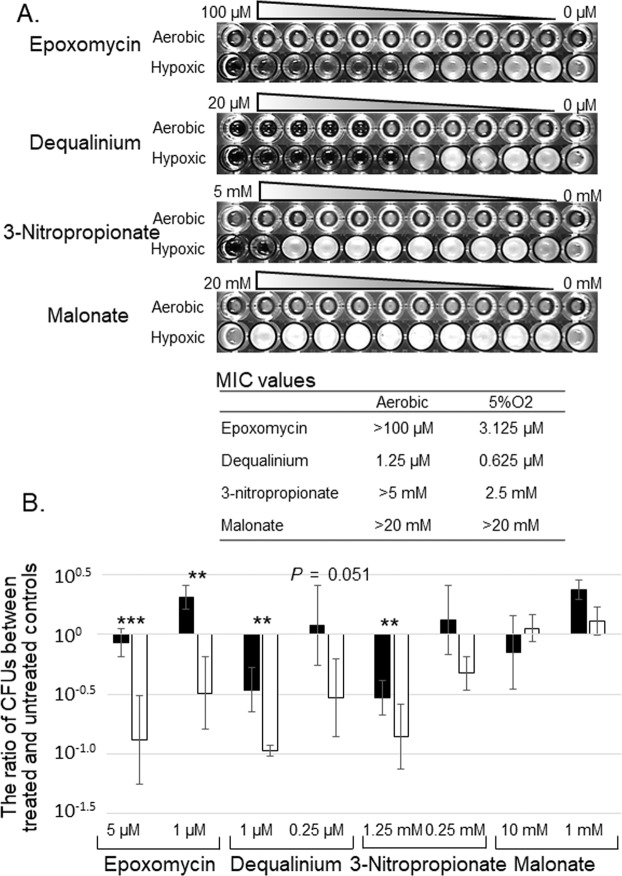
Comparison of the bacterial growth between aerobic and hypoxic conditions in the presence of metabolic inhibitors. Epoxomycin, dequalinium and 3-nitropropionate were used as inhibitors of the TnSeq-hit pathways, and malonate was used as an inhibitor of the pathways hit as a fitness advantage in TnSeq. (**A**) Minimum inhibitory concentration (MIC) assay. The MIC values are shown in the table. (**B**) Comparison of the ratio of colony forming units (CFUs) between aerobic and hypoxic conditions. The assay was performed in the presence of sub-MIC concentrations of inhibitors for aerobic (solid bars) and hypoxic conditions (open bars). The ratio was calculated as a difference of CFUs at Day 14 and CFUs at Day 0 divided by CFUs at Day 0. **P <  0.01; ***P <  0.001; unpaired two-tailed *t-*tests.

To validate the role of the genes showing fitness costs under hypoxia, we compared the minimum inhibitory concentration (MIC) values of several kinds of metabolic inhibitors between aerobic and hypoxic conditions. We found that the MIC values of proteasome inhibitor epoxomycin^38^, mycothiol metabolism inhibitor dequalinium^45^ and iscoitrate lyase inhibitor 3-nitropropionate^29,32^ were lower in hypoxic conditions than in aerobic conditions (Figs. 5, S3). A decrease in bacterial amount up to the order of 1-log_10_ was observed in the presence of sub-MIC concentrations of these metabolic inhibitors under hypoxic but not aerobic conditions. The inhibitor of succinate dehydrogenase malonate, as a negative control which showed a fitness advantage under hypoxia, did not inhibit growth in aerobic or hypoxic conditions. Furthermore, pellicle formation under hypoxia was significantly impaired in the presence of sub-MIC concentrations of the above-mentioned metabolic inhibitors on the TnSeq-hit pathways (Fig. S4). The higher effectiveness of inhibitors targeting genes with decreased fitness under hypoxia supports the biological relevance of these genes in maintaining survival under hypoxia for pellicle formation.

## Discussion

TnSeq analysis has been used to study various genera of bacteria^7^. In mycobacteria, essential genes for growth were determined globally in *M. tuberculosis* H37Rv^10–12^ and *M. marinum* E11^15^. We determined the essential genes of one of the human pathogenic NTM *M. intracellulare*. Targets of several kinds of antituberculous drugs were included in the essential genes common to *M. intracellulare* ATCC13950, *M. tuberculosis* H37Rv and *M. marinum* E11. Although the effect of chemotherapy is poorer in NTM disease than in drug-sensitive tuberculosis^46^, it is plausible that combination therapies using rifampin, ethambutol and levofloxacin could be clinically effective for treating infections by *M. intracellulare*, given the essentiality of the genes that are the targets of these drugs based on the TnSeq data. Interestingly, the trehalose glycolipid transporter gene *mmpL3*,which is the target of SQ109 currently in phase 2 of clinical trials for tuberculosis, was shown to be essential in *M. intracellulare* ATCC13950^47^. Although the relationship between *mmpL3* mutation and resistance to SQ109 was only demonstrated in *M. smegmatis* and *M. tuberculosis*^48^, the essentiality of *mmpL3* gene in *M. intracellualre* ATCC13950 suggests it is worth exploring for use against NTM.

Compared to the previous report by Chen^49^, the MIC values in *M. intracellulare* were comparatively higher than in *M. tuberculosis*, especially in isoniazid and ethionamide. Possible reason for the high MIC values in isoniazid and ethionamide may be they are prodrugs whose active form is produced by some enzymatic reaction (for example, catalase) inside bacteria^50^. Thus, not only the gene essentiality but also the activity of such enzymes can determine the effect of these drugs. As a result, the association of the effect of these prodrugs with the gene essentiality may be diminished by the activity of such enzymes in some degrees. Meanwhile, from the MIC data of the other antibiotics tested in this study (Table 2), the gene essentiality seems to be associated with the effect of the antibiotics overall. Therefore, the effectiveness of these drugs *in vitro* supports the idea of gene essentiality identified by TnSeq.

In *M. intracellulare*, the type VII secretion protein gene of ESX-5 *eccC5* was shown to be non-essential in the Tn mutant libraries, in contrast to the findings of previous studies that showed the essentiality of *eccC5* in *M. tuberculosis* and *M. bovis*^51^. Considering that increasing the permeability of the outer membrane in *M. marinum* mutants of phthiocerol dimycocerosate/phenolic glycolipid biosynthesis genes rescues the essentiality of ESX-5^51^, the essentiality of the ESX-5 system may be determined by the content of major glycolipids in the outer membrane. In *M. intracellulare*, the major glycolipid of the outer membrane is glycopeptidolipid, an amphiphilic molecule specific to MAC and *M. scrofulaceu*m group^52^. The difference of gene essentiality in the ESX-5 system between *M. intracellulare*, *M. marinum* and *M. tuberculosis* may be associated with the difference of the lipophilicity of the outer membrane depending on the component of major glycolipids.

Mycobacterial lesions are considered to be hypoxic as suggested by the hypoxia in tuberculous granuloma and inside biofilms^19,21^. This prompts us to consider functions that are essential under hypoxia could represent novel targets for drug discovery. We found differences in the required genes between aerobically-cultured planktonic bacteria and hypoxically-cultured pellicle bacteria, which reflects the metabolic shift, especially in carbohydrate metabolism. We demonstrated the requirement of the metabolic shift in the carbon metabolism for hypoxic pellicle formation in NTM for the first time (Fig. 4). Gluconeogenesis has been suggested to be important for displaying virulence by using knockout mutants of isocitrate lyase, phosphoenolpyruvate carboxykinase and glycogen biosynthesis genes in *M. tuberculosis*^29,31,32,53^. We found several genes of rate-limiting gluconeogenesis enzymes (fructose-1,6-bisphosphatase, phosphofructokinase and phosphoenolpyruvate carboxykinase) and glycogen synthase gene to be required under hypoxia. The essentiality of gluconeogenesis for hypoxic pellicle formation is consistent with a previous report in *M. tuberculosis* showing that disruption of phosphoenolpyruvate carboxykinase (*pck*) gene leads to the blocking of gluconeogenesis metabolically^31^.

In *M. tuberculosis*, two distinct components of the TCA cycle are known to be utilized from α-ketoglutarate: one is α-ketoglutarate decarboxylase and the other is α-ketoglutarate:ferredoxin oxidoreductase^54,55^. The former functions in the absence of β-oxidation and the latter functions concurrently with β-oxidation, resulting in completion of the oxidative TCA cycle from α-ketoglutarate^54^. Consistent with the importance of succinate production in adaptation to hypoxia previously shown in metabolomics study in *M. tuberculosis*^30^, both α-ketoglutarate decarboxylase and α-ketoglutarate:ferredoxin oxidoreductase were required under hypoxia in *M. intracellulare*. Moreover, the serine/threonine protein kinase PknG is known to act as a sensor of glutamine and glutamate level thorough phosphorylation of the effector molecule, GarA, which degrades glutamate by activating glutamate dehydrogenase^39^. α-ketoglutarate decarboxylase and α-ketoglutarate:ferredoxin oxidoreductase catabolize glutamate in combination with glutamate dehydrogenase. Such adaptation of carbon metabolism including gluconeogenesis and the TCA cycle may therefore be speculated to confer hypoxic pellicle formation in NTM.

Our result that the inhibitors of the proteasome system epoxomycin and mycothiol redox system dequalinium suppress bacterial growth more effectively under hypoxia than aerobic conditions supports the previous finding of the involvement of these systems in virulence *in vivo*. The knockout mutants of proteasome genes have been shown to be more sensitive to nitrosative stress and display impaired growth in mice^38^. A principal substrate of the proteasome system has been identified as malonyl-CoA acyl carrier protein transacylase (FabD) and ketopantoate hydroxymethyltransferase (PanB), both of which are required for biosynthesis of fatty acids and polyketides^56^. In addition, the requirement of some of the proteasomal genes has been suggested by TnSeq analysis when *M. tuberculosis* replicates within dendritic cells^57^. Mycothiol is a mycobacterium-specific thioredox system and the redox potential is maintained by the previously-mentioned PknG-GarA system under hypoxic conditions as well as various kinds of stress such as oxidative damage and antibiotic exposure^27,28^. These mycobacterium-specific metabolic pathways may have a role in maintaining persistent mycobacterial infections and thus, they may be promising targets for discovery of antibiotics specific to mycobacteria.

The genes shown to be essential with decreased fitness under hypoxia (Tables S7, S9) may also play a role in the virulence of NTM. Phosphatidylinositol metabolism confers the production of lipoarabinomannan, one of the major cell wall glycolipids, and it confers acid-fastness, resistance to β-lactams and virulence in macrophages and in mice^36^. The requirement of *pimE* under hypoxia may reflect the suppression of inflammatory response and killing by macrophages as suggested by previous studies in *M. avium*^37^. Furthermore, information on mycobacteria-specific secretion/transport system, such as ESX and MmpL, has been increasing recently. ESX-5 has been shown to be involved in the uptake of fatty acids^43^. In addition, the uptake of iron may confer a fitness advantage under hypoxia as estimated by the hit data of ESX-3 and some heme transporter homologous to *mmpL11*^41,42,58^. These data support the role of cell wall glycolipid and transport of fatty acids and iron to form a pellicle as an adaptation to hypoxia.

Several reports showed pellicle or pellicle-like biofilm formation in *M. tuberculosis* and *M. smegmatis*^59–62^. However, they identified *groEL1*, *mutT3* (neighboring *pknG*), and malate dehydrogenase as genes that confer biofilm formation, which is contrary to our study results that identified them as non-essential with no significant fitness change under hypoxia. The reason for this inconsistency may be due to the difference in experimental models between the studies. For example, the key condition for pellicle formation in *M. intracellulare* is hypoxia and eutrophy used in our study, not aerobic incubation in oligotrophy or exogenous reductive stress by dithiothreitol. As such, it may be recommended to avoid simple extrapolation of *M. tuberculosis* and *M. smegmatis* findings to NTM.

There are some limitations in this study. First, there is a difference in the shared proportion of essential genes between *M. tuberculosis* H37Rv (55.3% of its essential genes) and *M. marinum* E11 (31.2% of its essential genes). This suggests the existence of mycobacterial species-specific essential genes. However, we cannot exclude the possibility of discrepancies due to each method. One reason may be due to the difference in the sequencing model of transposon mutant libraries. The *M. tuberculosis* H37Rv data were sequenced by the same model used in our study (PCR amplification of junctional sequence followed by HMM analysis)^11^. The *M. marinum* E11 data were sequenced by TraDIS (a similar method to ours except with fewer cycles [22 cycles] of PCR amplification of transposon junction, without adding the adaptor and index sequences, followed by custom-made computer analysis)^15^. Another reason might be the difference in the quality of annotation between manual curation for *M. tuberculosis* H37Rv and automated annotation for *M. intracellulare* ATCC13950. The latter tends to produce short genes, most of which are annotated as hypothetical. Such short genes may increase the number of essential genes detected by TnSeq. To increase the certainty of TnSeq data, genome annotation data must be improved.

Second, the essential genes for pellicle formation included both sets of genes that are critical for survival under hypoxia and those that are critical for the process of building-up a pellicle, for example clustering bacterial cells, microcolony formation, and production of the extracellular matrix. Further study is necessary to elucidate the precise role of each gene for long-term survival of NTM using various experimental settings such as hypoxic growth unable to form a pellicle by shaking or stirring the culture.

In conclusion, by utilizing functional genomic analysis of diverse atmospheric conditions in NTM, we identified a wide range of metabolic pathways including targets of antituberculous drugs, mycobacterial virulence-associated factors and central carbon metabolism as essential for growth and survival. The functional genomic data including conditions relevant to the host environment provide an additional viewpoint to find novel targets for drug discovery in NTM.

## Materials and Methods

### Bacterial strains and phages

*M. intracellulare* ATCC13950 was used as a parental strain for construction of the transposon mutant libraries. Mycobacterial phage phAE180^63^ (gifted from Dr. William R. Jacobs, Jr., Albert Einstein College of Medicine, NY, USA) was used for transducing transposon into the ATCC13950 bacterial cells. The backbone of phAE180 is a temperature-sensitive bacteriophage TM4^64^ and phAE180 has a mariner-class transposon element (*Himar1*) identified from horn flies *Haematobia irritans*^65^.

### Transposon mutagenesis and preparation of bacterial samples for TnSeq

In this study, three Tn mutant libraries of *M. inracellulare* ATCC13950 were prepared independently (Fig. 1). *M. intracellulare* mariner-based transposon mutagenesis was carried out as previously described^63^. Wild-type *M. inracellulare* ATCC13950 was inoculated in Middlebrook 7H9 medium (Difco Laboratories, Detroit, MI) containing 0.2% glycerol, 0.1% Tween 80 (MP Biomedicals, Illkirch, France) and 10% Middlebrook albumin-dextrose-catalase (7H9/ADC/Tween 80) without antibiotics. One hundred milliliter of broth cultures were grown to optical densities around 1.0. After harvesting the bacteria by centrifugation, 10 ml of high titer MycoMarT7 phagemids (>1 × 10^9^ PFU/ml) suspended in MP buffer (50 mM Tris-HCl [pH 7.5], 150 mM NaCl, 2 mM CaCl_2_) was transduced at 37 °C overnight. After centrifugation, the harvested bacteria were resuspended in 10 ml of 7H9/ADC/Tween 80, inoculated on Middlebrook 7H10 solid medium supplemented with 10% oleic acid-albumin-dextrose-catalase (Becton Dickinson and Co., Sparks, MD) (7H10/OADC) agar plates containing 25 μg/ml kanamycin, and cultured at 37 °C for 2 weeks. The kanamycin-resistant colonies (>1 × 10^5^) were evenly resuspended in 7H9/ADC containing 20% glycerol, aliquoted and stored at −80 °C until further use.

In this study, the cultures of planktonic and pellicle bacteria were prepared from each replicate of the Tn mutant libraries mentioned above (Fig. 1). The protocol of planktonic and pellicle bacterial culture was followed from the previous report^16^. To obtain planktonic bacteria, we started 100 ml cultures from OD 0.003 in 7H9/ADC (without Tween 80) and incubated aerobically in static conditions at 37 °C for 1 week. To obtain pellicle bacteria, we started 3 ml/well cultures from OD 0.003 in 12-well plates and incubated statically in 5% oxygen conditions at 37 °C for 3 week. Planktonic bacteria were harvested by centrifugation (2,300 × *g* for 30 min) followed by washing by distilled water twice. Pellicle bacteria were harvested by scraping the pellicle formed on the air-liquid interface, followed by washing with distilled water twice. Genomic DNA was extracted by phenol-chloroform method^66^.

### TnSeq

The TnSeq libraries were constructed as previously described^12^. The resultant TnSeq libraries were sequenced using a HiSeq. 2500HO, 125 bp PE run using v4 chemistry (Illumina). Sequence reads were analyzed as previously described^12^. Briefly, we first trimmed sequence reads for transposon and adaptor sequences and then discarded the sequence reads that were shorter than 18 bp. We used the CutAdapt^67^ default error rate of 0.1 for all trimming processes. The trimmed sequence reads were mapped (allowing 1 bp mismatch) to the *M. intracellulare* ATCC13950 genome (GenBank: NC_016946.1) and converted output to SAM format using Bowtie2^68^. The numbers of sequence reads at each TA site were counted and converted to the wig format, the input file format for TRNSIT^23^ using the custom Python script^12^. After averaging the obtained read counts between the three replicates in each experimental setting, statistical analysis for determination of essential genes and fitness for hypoxic growth was performed by the Hidden Markov Model (HMM) and resampling analysis on TRANSIT, respectively.

### Comparative genomics

To identify the homologous genes of *M. intracelluare* ATCC13950 with *M. tuberculosis* H37Rv (GenBank: NC_000962.3) and *M. marinum* E11 (GenBank: NZ_HG917972.2), reciprocal BLAST search was performed by using GView Server with expected cutoff, alignment length cutoff and percent identity cutoff as 1.0E-10, 100 and 80, respectively^69^. The UpSet plot was drawn by using the UpSetR package in R.

### Pathway analysis

The list of genes hit by resampling analysis was submitted to the KEGG database through BLASTKoala. The required pathways for pellicle formation were identified from the BRITE reconstruction result.

### Evaluation of the effect of antituberculous drugs and metabolic inhibitors on bacterial growth

SQ109 was kindly provided by Dr. Helena Boshoff (National Institute of Health, USA). Other antituberculous drugs used in this study were purchased from FUJIFILM Wako Pure Chemical (Osaka, Japan) and Tokyo Chemical Industry (Tokyo, Japan). The MICs of these drugs were measured by inoculating the bacteria (OD_600_ 0.003) with serially-diluted inhibitors in 96-well plates followed by incubation at 37 °C for 2 weeks. Epoxomycin and malonate were purchased from Peptide Institute (Osaka, Japan) and FUJIFILM Wako Pure Chemical, respectively. Dequalinium and 3-nitropropionate were purchased from Sigma Aldrich (St. Louis, USA). To evaluate the effect of inhibiting the TnSeq-hit pathways on bacterial growth, the MIC values of these metabolic inhibitors were compared between aerobic and hypoxic conditions at 2 weeks. Besides, planktonic bacterial growth in glass tubes was evaluated by colony forming unit (CFU) assay and hypoxic bacterial growth (pellicle formation) in glass tubes was evaluated by CFU assay as well as pellicle thickness at 2 weeks. Data of pellicle thickness were evaluated by unpaired *t*-test and the significance level was set as *P* < 0.05.

## Supplementary information

Supplementary information.

Additional file 3.

Additional file 4.

Additional file 5.

Additional file 8.

Additional file 9.

Additional file 10.

Additional file 11.

## Data Availability

The datasets used/or analyzed during the current study are available from the corresponding author on reasonable request.
