# Supplementary material for: Genome-wide identification of essential genes in Mycobacterium intracellulare by transposon sequencing — Implication for metabolic remodeling
Source: Sci Rep. 2020 Mar 25;10:5449. doi: 10.1038/s41598-020-62287-2 (PMC7096427; doi:10.1038/s41598-020-62287-2)
Supplement: Supplementary file 1 — Supplementary information. [file 41598_2020_62287_MOESM1_ESM.pdf]

Supplementary information for "Genome-wide identification of essential genes in *Mycobacterium intracellulare* by transposon sequencing — Implication for metabolic remodeling"

Authors

Yoshitaka Tateishi[1*], Yusuke Minato[2], Anthony D. Baughn[2], Hiroaki Ohnishi[3], , Akihito Nishiyama[1], Yuriko Ozeki[1], Sohkichi Matsumoto[1]

1. Department of Bacteriology, Graduate School of Medical and Dental Sciences, Niigata University, 1-757, Asahimachi-Dori, Chuo-ku, Niigata, 951-8510, Japan.
2. Department of Laboratory Medicine, Kyorin University School of Medicine, Tokyo, Japan.
3. Department of Microbiology and Immunology, University Minnesota Medical School, 689 23rd Avenue S.E. Microbiology Research Facility, Minneapolis MN 55455.

* Corresponding author (Correspondence to y-tateishi@med.niigata-u.ac.jp)

Legends of supplementary data

Additional file 1: Figure S1. Pellicle formation in *M. intracellulare* ATCC13950 under hypoxic conditions. (A)Photographs of aerobically-cultured planktonic bacteria and hypoxically-cultured pellicle bacteria. Pellicles were specifically formed in hypoxic conditions (5% oxygen). (B) Time-course change of pellicle thickness cultured under hypoxic conditions. The data were obtained from three independent assays (mean ± SD).

Additional file 2: Figure S2. Overview of the transposon (Tn) insertion data in aerobically-cultured planktonic bacteria (PLK) and hypoxically-cultured pellicle bacteria (PEL). (A) Distribution and density of Tn insertion on the ATCC13950 genome. Tn insertion reads (vertical black bars) are shown in the upper row. Coding sequence (CDS) (red), %GC plot (black, upward = above average, downward = below average) and GC skew (purple) are shown in the lower row. (B) Sequencing reproducibility. The graph compares the number of insertions per gene between batches of PLK and PEL, respectively.

Additional file 3: Table S1. Essential genes of *M. intracellulare* ATCC13950 Tn mutant library bacteria.

Additional file 4: Table S2. Number of Tn insertion sites hit per gene in the *M. intracellulare* ATCC13950 Tn mutant library bacteria.

Additional file 5: Table S3. List of essential genes of *M. intracellulare* ATCC13950 shared with those of *M. tubervulosis* H37Rv and *M. marinum* E11.

Additional file 6: Table S4. Homologs of essential genes of *M. intracellulare* ATCC13950 in *M. tuberculosis* H37Rv and *M. marinum* E11 corresponding to existing antituberculous drug targets.

Additional file 7: Table S5. Result of next-generation sequencing and mapping data of aerobically-cultured planktonic bacteria (PLK) and hypoxically-cultured pellicle bacteria (PEL).

Additional file 8: Table S6. Specific essential genes of *M. intracellulare* ATCC13950 under hypoxia.

Additional file 9: Table S7. Essential genes specific to hypoxic conditions that showed significant decrease of fitness compared to aerobic conditions.

Additional file 10: Table S8. Genes showing fitness change under hypoxia compared to aerobic conditions.

Additional file 11: Table S9. Representative virulence-associated genes showing decreased fitness for hypoxic survival to form pellicles.

Additional file 12: Figure S3. Interpretation of MIC data in PLK bacteria and PEL bacteria. The PLK bacteria sink on the bottom of the round-bottom plate. The PLK bacteria can be observed as a circular dot in the center of the well. The PEL bacteria form a membranous pellicle floating on the air-liquid interface. The PEL bacteria can be observed as covering all over the well.

Additional file 13: Figure S4. Effect of metabolic inhibitors of the TnSeq-hit pathways on pellicle formation under hypoxia. (A) View of the culture in glass tubes in aerobic and hypoxic conditions. (B) Quantification of pellicle thickness cultured under hypoxia. The significant difference compared to no drug control was shown by an asterisk. Pellicles were not formed under aerobic conditions.

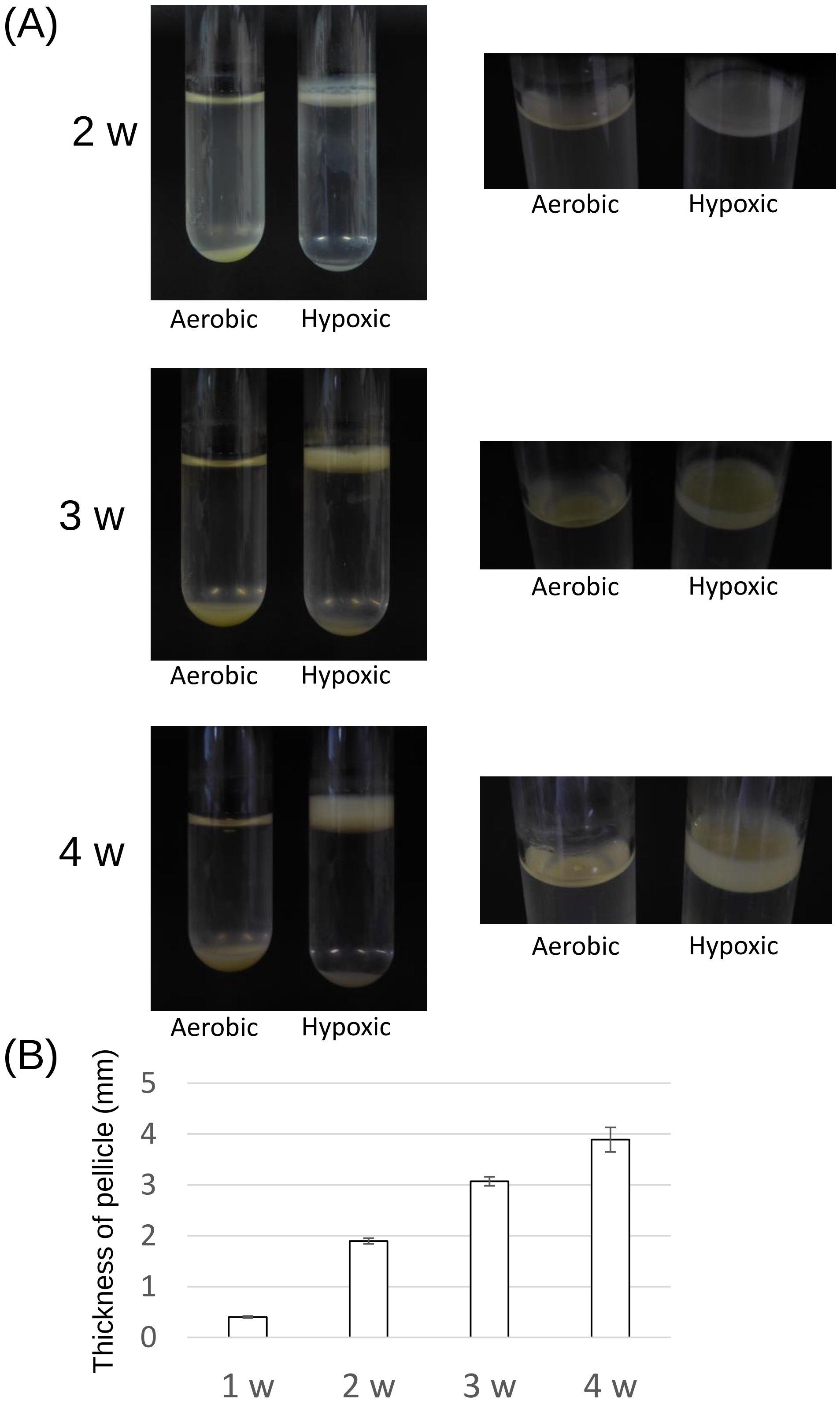
(A)
2 w
Aerobic
Hypoxic
Aerobic
Hypoxic
3 w
Aerobic
Hypoxic
Aerobic
Hypoxic
4 w
Aerobic
Hypoxic
Aerobic
Hypoxic
(B)
Thickness of pellicle (mm)
0
1
2
3
4
5
1 w
2 w
3 w
4 w

Fig S1

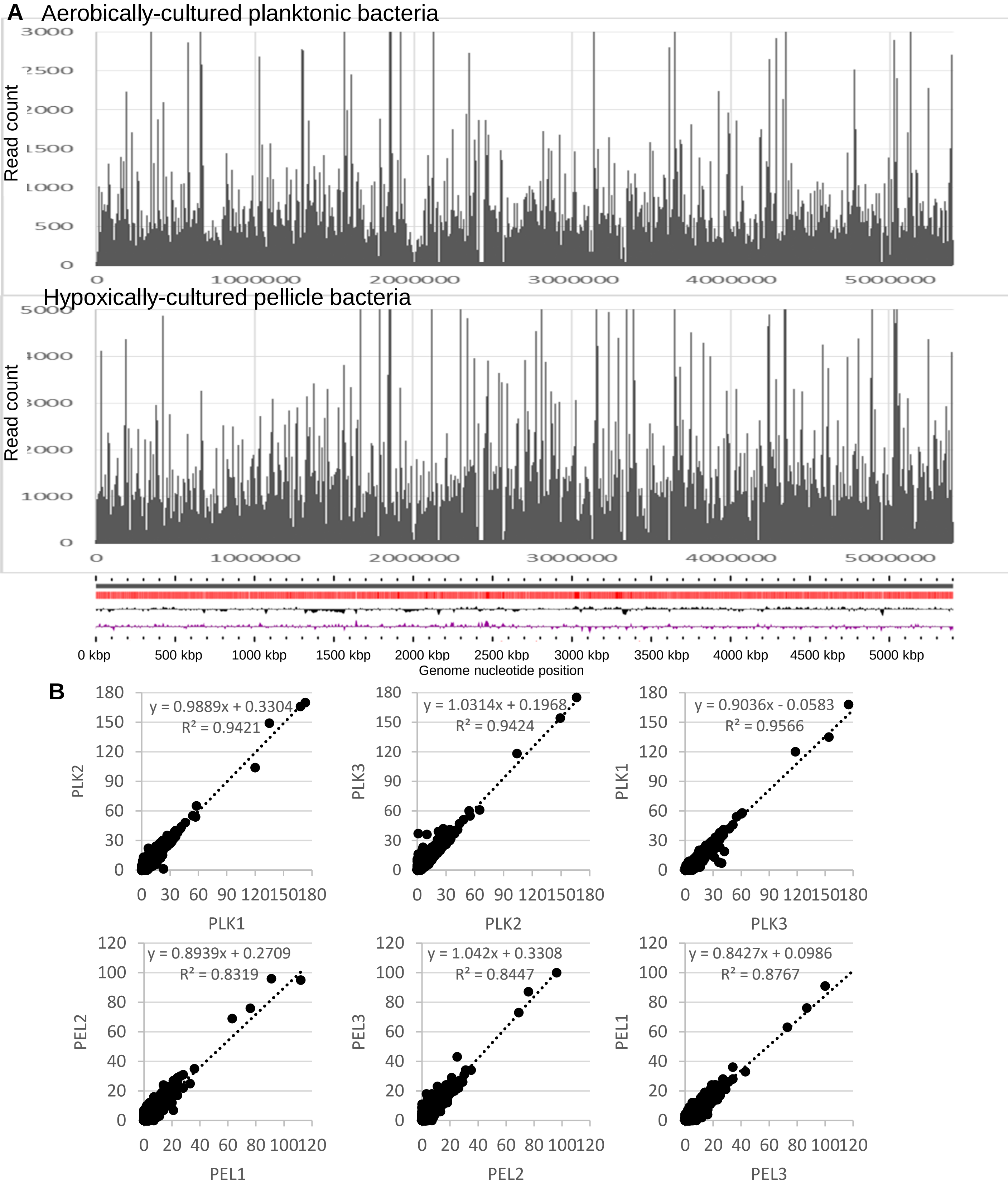
A
Aerobically-cultured planktonic bacteria
Read count
0
500
1000
1500
2000
2500
3000
0
1000000
2000000
3000000
4000000
5000000
Hypoxically-cultured pellicle bacteria
Read count
0
1000
2000
3000
4000
5000
0 kbp
500 kbp
1000 kbp
1500 kbp
2000 kbp
2500 kbp
3000 kbp
3500 kbp
4000 kbp
4500 kbp
5000 kbp
Genome nucleotide position
B
y = 0.9889x + 0.3304
R² = 0.9421
PLK2
PLK1
y = 1.0314x + 0.1968
R² = 0.9424
PLK3
PLK2
y = 0.9036x - 0.0583
R² = 0.9566
PLK1
PLK3
y = 0.8939x + 0.2709
R² = 0.8319
PEL2
PEL1
y = 1.042x + 0.3308
R² = 0.8447
PEL3
PEL2
y = 0.8427x + 0.0986
R² = 0.8767
PEL1
PEL3

Fig S2

Table S4. Homologs of essential genes of *M. intracellulare* ATCC13950 in *M. tuberculosis* H37Rv and *M. marinum* E11 corresponding to existing antituberculous drug targets.

Gene locus	Gene	Function	Homolog in *M. tuberculosis* H37Rv	Homolog in *M. marinum* E11 (designated as locus tag)	Target drug
OCU_RS25020	*gyrB*	DNA topoisomerase (ATP-hydrolyzing) subunit B	*rv0005*	MMARE11_00050	fluoroquinolone
OCU_RS25025	*gyrA*	intein-containing DNA gyrase subunit A	*rv0006*	MMARE11_00060	fluoroquinolone
OCU_RS26150	*embB*	arabinosyltransferase	*rv3795*	MMARE11_51780	ethambutol
OCU_RS26155	*embA*	arabinosyltransferase	*rv3794*	MMARE11_51770	ethambutol
OCU_RS26175	*embC*	arabinosyltransferase	*rv3793*	MMARE11_51760	ethambutol
OCU_RS40185	*inhA*	enoyl-[acyl-carrier-protein] reductase FabI	*rv1484*	MMARE11_22120	isoniazid/ ethionamide
OCU_RS41875	*dfrA*	dihydrofolate reductase	*rv2763c*	NH	para aminosalicylic acid
OCU_RS45570	*alr*	alanine racemase	*rv3423c*	MMARE11_10770	cycloserine
OCU_RS46230	*rpoB*	DNA-directed RNA polymerase subunit β	*rv0667*	MMARE11_09460	rifampin
OCU_RS48685	*mmpL3*	membrane protein mmpL3	*rv0206c*	MMARE11_04100	SQ109

NH: no homology more than 80% of percent identity of nucleotide sequence

Table S5. Result of next-generation sequencing and mapping data of planktonic bacteria (PLK) and pellicle bacteria (PEL)

Library	No. of sequence reads	No. (%) of aligned reads	No. (%) of insertion site flanking sequences hit in library	Average no. of reads/flanking sequence
PLK1	10,932,182	5,539,472 (54.9%)	38,430 (59.8%)	201
PLK2	8,579,984	5,175,328 (64.3%)	34,356 (53.4%)	234
PLK3	7,817,364	4,700,645 (64.2%)	36,225 (56.3%)	217
PEL1	9,271,142	5,355,509 (61.7%)	18,270 (28.4%)	491
PEL2	9,634,505	5,570,149 (61.4%)	18,142 (28.2%)	532
PEL3	9,914,886	5,542,869 (59.8%)	16,595 (25.8%)	506

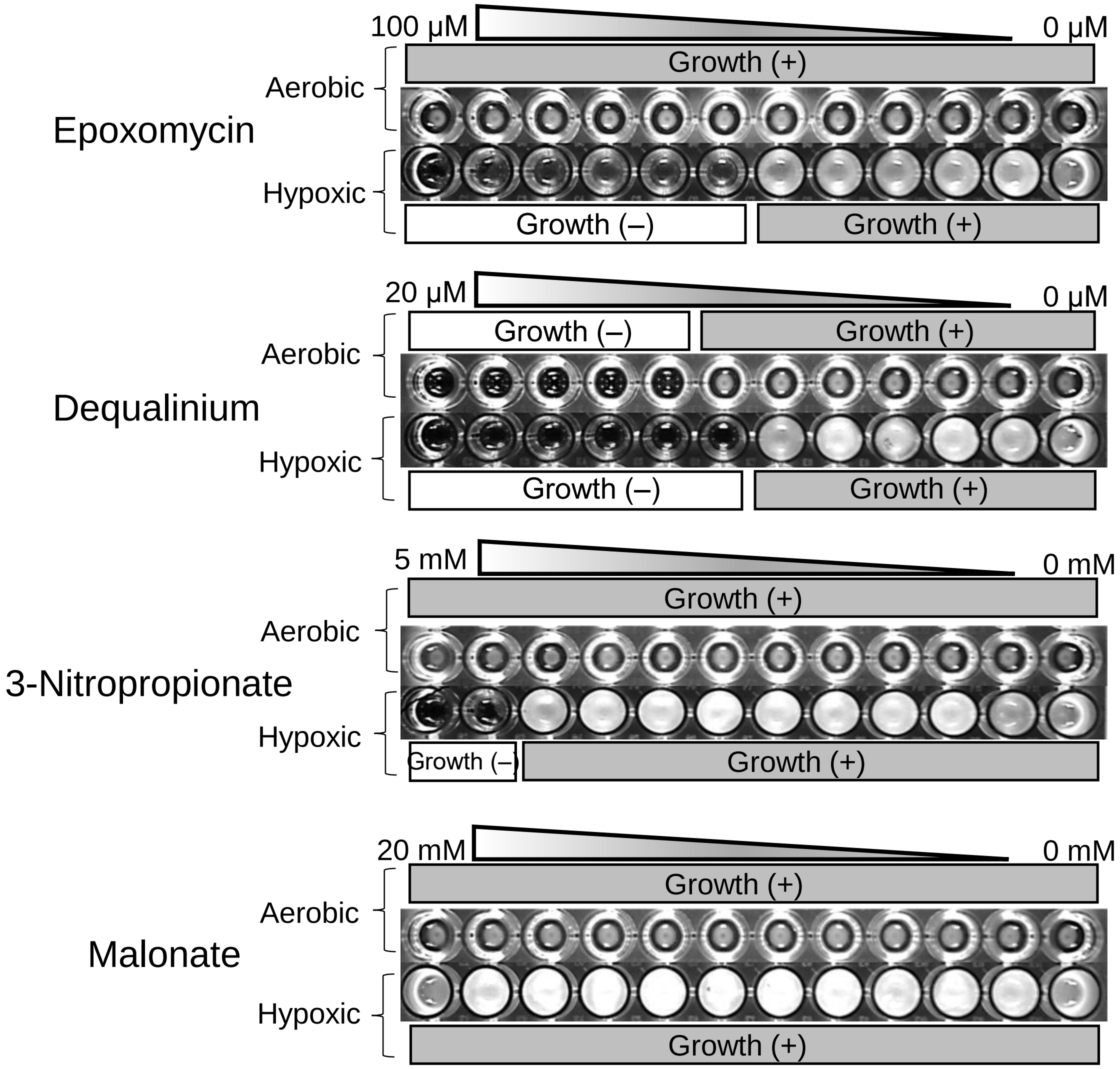

100 μM
0 μM
Growth (+)
Aerobic
Epoxomycin
Hypoxic
Growth (–)
Growth (+)
20 μM
0 μM
Growth (–)
Growth (+)
Aerobic
Dequalinium
Hypoxic
Growth (–)
Growth (+)
5 mM
0 mM
Growth (+)
Aerobic
3-Nitropropionate
Hypoxic
Growth (–)
Growth (+)
20 mM
0 mM
Growth (+)
Aerobic
Malonate
Hypoxic
Growth (+)

Fig S3

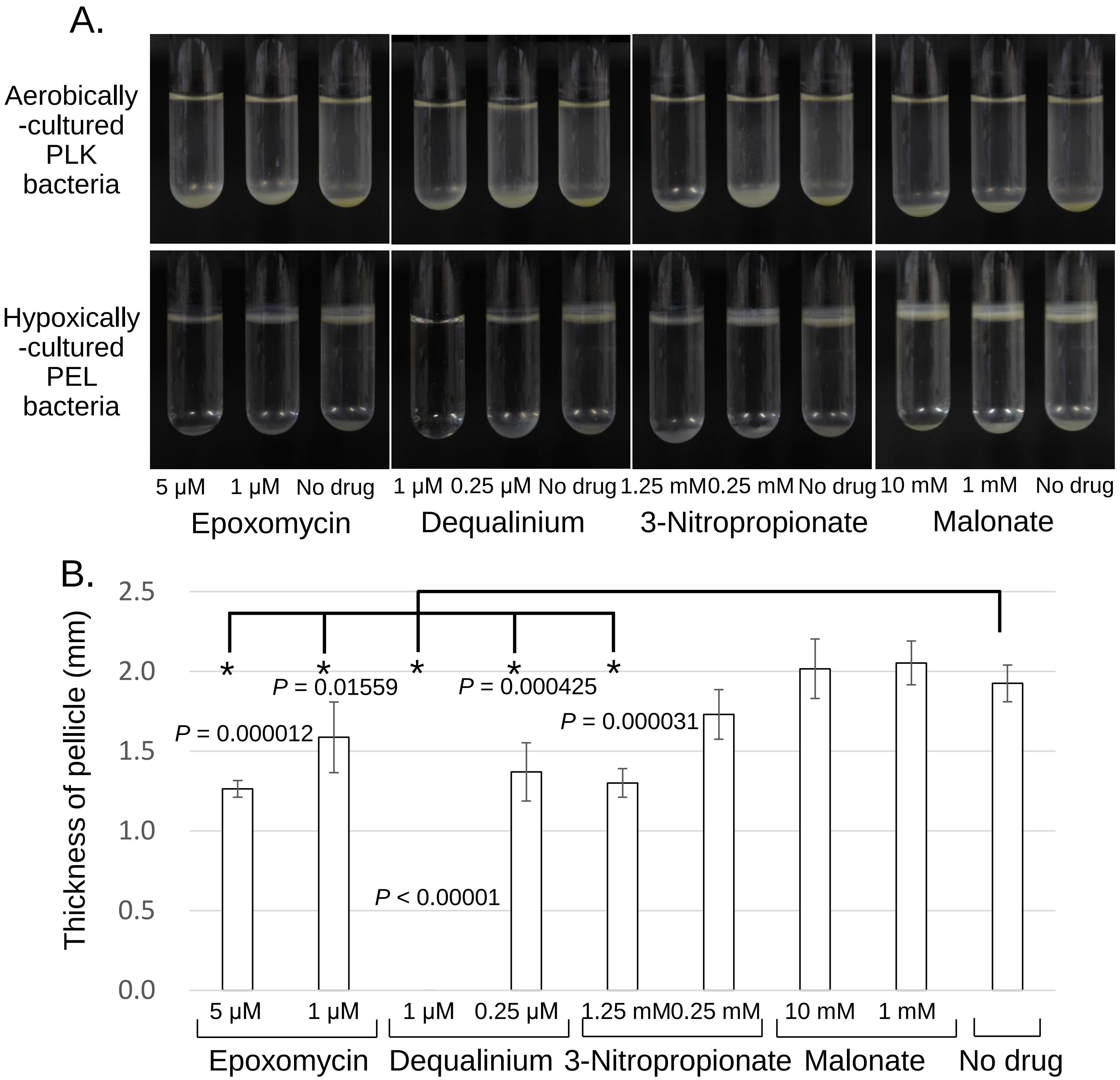
A.
Aerobically -cultured PLK bacteria
Hypoxically -cultured PEL bacteria
5 μM
1 μM
No drug
1 μM
0.25 μM
No drug
1.25 mM
0.25 mM
No drug
10 mM
1 mM
No drug
Epoxomycin
Dequalinium
3-Nitropropionate
Malonate
B.
Thickness of pellicle (mm)
2.5
2.0
1.5
1.0
0.5
0.0
*
P = 0.000012
P = 0.01559
P < 0.00001
P = 0.000425
P = 0.000031
5 μM
1 μM
1 μM
0.25 μM
1.25 mM
0.25 mM
10 mM
1 mM
Epoxomycin
Dequalinium
3-Nitropropionate
Malonate
No drug

Fig S4
